# Synaptic properties of mouse tecto-parabigeminal pathways

**DOI:** 10.3389/fnsys.2023.1181052

**Published:** 2023-05-12

**Authors:** Kyle L. Whyland, Sean P. Masterson, Arkadiusz S. Slusarczyk, Martha E. Bickford

**Affiliations:** Department of Anatomical Sciences and Neurobiology, School of Medicine, University of Louisville, Louisville, KY, United States

**Keywords:** superior colliculus, tectum, parabigeminal nucleus, nucleus isthmi, electron microscopy, optogenetics, GABA, parvalbumin

## Abstract

The superior colliculus (SC) is a critical hub for the generation of visually-evoked orienting and defensive behaviors. Among the SC’s myriad downstream targets is the parabigeminal nucleus (PBG), the mammalian homolog of the nucleus isthmi, which has been implicated in motion processing and the production of defensive behaviors. The inputs to the PBG are thought to arise exclusively from the SC but little is known regarding the precise synaptic relationships linking the SC to the PBG. In the current study, we use optogenetics as well as viral tracing and electron microscopy in mice to better characterize the anatomical and functional properties of the SC-PBG circuit, as well as the morphological and ultrastructural characteristics of neurons residing in the PBG. We characterized GABAergic SC-PBG projections (that do not contain parvalbumin) and glutamatergic SC-PBG projections (which include neurons that contain parvalbumin). These two terminal populations were found to converge on different morphological populations of PBG neurons and elicit opposing postsynaptic effects. Additionally, we identified a population of non-tectal GABAergic terminals in the PBG that partially arise from neurons in the surrounding tegmentum, as well as several organizing principles that divide the nucleus into anatomically distinct regions and preserve a coarse retinotopy inherited from its SC-derived inputs. These studies provide an essential first step toward understanding how PBG circuits contribute to the initiation of behavior in response to visual signals.

## Introduction

Growing evidence has implicated the superior colliculus (SC) as a critical hub for the generation of appropriate actions in response to visual stimuli (Shang et al., 2015; Wei et al., 2015; Zingg et al., 2017; Masullo et al., 2019). The SC is well suited for this role, given that its superficial layers receive abundant input from the retina, while premotor neurons reside in its deeper layers (Sahibzada et al., 1986; Ellis et al., 2016; Masullo et al., 2019; Basso et al., 2021). Retinal input to the SC is organized in a retinotopic fashion, creating a topographic representation of visual space across its superficial layers (Sparks and Mays, 1980; Chandrasekaran et al., 2005), while the deeper layers contain motor or priority maps (Robinson, 1972; Schiller and Stryker, 1972; Sparks, 1978). This organization allows for specialized populations of neurons within the SC to form discreet functional modules to direct motor responses to visual stimuli in a targeted fashion (Masullo et al., 2019). In addition, the visual layers of the SC target many extrinsic structures which can also trigger visually-evoked movements. These targets include the parabigeminal nucleus (PBG), a small group of neurons on the lateral wall of the midbrain (Graybiel, 1978). The PBG, sometimes referred to as the “satellite of the superior colliculus,” receives input from the superficial visual layers of the SC and projects to multiple visuomotor structures including the dorsal lateral geniculate nucleus, pulvinar, central amygdala and superficial layers of the SC (Graybiel, 1978; Sherk, 1979b; Usunoff et al., 2007; Shang et al., 2015; Whyland et al., 2020; Sokhadze et al., 2022). Recent studies indicate that activation of SC inputs to the PBG contributes to the production of visually-evoked defensive behaviors (Shang et al., 2015, 2018; Zingg et al., 2017). Moreover, perturbing the zebrafish nucleus isthmi (NI), considered to be the PBG homolog, disrupts behavioral action selection (Fernandes et al., 2021).

Across species, recordings from PBG neurons indicate that they exhibit robust visual responses. In the cat, PBG neurons were found to respond to input from either eye, and exhibit velocity tuning and direction selectivity (Sherk, 1978, 1979a). In the mouse, PBG neurons also exhibit direction-selectivity and additionally are responsive to looming (rapidly expanding) and sweeping (small, fast moving) visual stimuli (Reinhard et al., 2019). Likewise, a population of neurons within the pigeon isthmic complex exhibit direction selectivity (Wang et al., 2022). Beyond simply encoding visual stimulus movement and direction, the PBG also appears to encode the saliency of visual stimuli. In cats, PBG cells respond to visual stimuli that predict the anticipated utility of saccade targets, supporting a potential role for the PBG in movement choice (Ma et al., 2013). Additionally, presentation of visual stimuli in one or both hemifields can either excite or inhibit neurons in the PBG, likely contributing to the binocular interactions necessary for proper visuomotor coordination across hemispheres of the brain (Ma et al., 2013). Finally, in zebrafish, the NI plays an essential role in weighing the saliency of competing visual stimuli, creating a contextually based mechanism for generating appropriate motor responses (Fernandes et al., 2021).

The response properties of neurons residing in the PBG appear to be inherited in large part from their SC input. First, in both cats and rodents, the PBG exhibits a rough retinotopic organization that mimics that found in the SC (Sherk, 1978, 1979b; Deichler et al., 2020). Second, the receptive field properties of SC and PBG neurons are similar in many respects (Sherk, 1979a). However, several pathways from the SC to the PBG have been identified which may be integrated in the nucleus to create unique response properties. One pathway originates from SC neurons that express parvalbumin (which may include narrow field vertical cells labeled in the Grp-KH288-cre line) and another pathway is composed of neurons that contain GABA but not parvalbumin (Gale and Murphy, 2014, 2018; Shang et al., 2015, 2018; Villalobos et al., 2018; Hoy et al., 2019; Whyland et al., 2020). These two pathways provide opposing influences on the activity of PBG neurons; NFV (narrow-field vertical) SC neurons labeled in the Grp-KH288-cre line excite the PBG, while GABAergic SC neurons inhibit the PBG (Gale and Murphy, 2014, 2018). Moreover, these pathways may provide the PBG with a variety of visual signals. NFV neurons labeled in the Grp-KH288 line exhibit small receptive fields, prefer small fast-moving stimuli, and are often direction-selective (Hoy et al., 2019). GABAergic SC neurons that project to the PBG can exhibit stellate, NFV or horizontal morphologies (Whyland et al., 2020); horizontal GABAergic SC neurons exhibit large receptive fields and are rarely direction-selective (Gale and Murphy, 2014, 2018), while the broader population of GABAergic neurons residing in the SGS, especially those in the most superficial SGS, generally exhibit direction-selectivity which is suppressed by surrounding motion in the opposite direction (Barchini et al., 2018).

How these SC signals are potentially integrated by PBG neurons is unknown. In the current study, we characterized the ultrastructure and synaptic properties of GABAergic and glutamatergic/parvalbumin SC inputs to the mouse PBG as well as the dendritic morphology of postsynaptic PBG neurons. We also identified non-tectal GABAergic input to the PBG, partially arising from neurons in and surrounding the PBG. These studies provide a critical first step toward uncovering how the PBG contributes to the initiation of visually-evoked behavior.

## Materials and methods

### Animals

All breeding and experimental procedures were approved by the University of Louisville Institutional Animal Care and Use Committee. Experiments were carried out using mice, of either sex, in C57BL/6J mice (Jax Stock No: 000664), a line in which neurons that contain the 67KD isoform of glutamic acid decarboxylase (GAD) express green fluorescent protein (GAD67-GFP; Jax Stock No: 007677, G42 line), a line in which neurons that contain GAD express cre-recombinase (GAD2-cre; Jax Stock No: 010802; GAD2), and a line in which neurons that contain parvalbumin (PV) express cre-recombinase (PV-cre; Jax Stock No: #008069).

### BDA and AAV virus injections

To label and/or optogenetically activate SC-PBG or tegmentum-PBG projections, Biotinylated dextran amine (BDA) or AAVs were injected bilaterally into the SC or the tegmentum of C57BL/6J, GAD2-Cre, or PV-Cre mice. The AAVs used for fluorescent labeling and activation of SC-PBG or tegmentum-PBG projections were: AAV1-CAG-Flex-EGFP-WPRE.bGH, AAV9-Flex-Rev-O-ChieftdTomato, AAV1-Acagw-O-Chieftdtom (which carries a vector for the Channelrhodopsin variant Chimera EF with I170 mutation [ChIEF] fused to the red fluorescent protein, tdTomato [for production details, see Jurgens et al., 2012]) or AAV5-Syn-Flex-rc[Chrimson-tdTomato]. In other experiments, to label SC-PBG projections for electron microscopy, BDA was injected unilaterally in the SC, or pENN.AAV1.hSyn.Cre.WPRE.hGH was injected in the eyes or the primary visual cortex (V1) to express cre-recombinase in SC neurons that receive input from the retina or V1, and the SC was subsequently injected bilaterally with AAV1-CAG-Flex-EGFP-WPRE.bGH. For adeno-associated (AAV) delivery in V1, tegmentum, or the SC, P21-P35 mice were deeply anesthetized with a mixture of ketamine (100–150 mg/kg) and xylazine (10–15 mg/kg). The analgesic meloxicam (1–2 mg/kg) was also injected prior to surgery. The animals were then placed in a stereotaxic apparatus (Angle Two Stereotaxic, Leica, Wetzlar, Germany). An incision was made along the scalp, and a small hole was drilled in the skull overlying the SC, tegmentum, or V1. Virus was infused into the brain via a 34-gauge needle attached to a Nanofil syringe inserted in an ultramicropump. A volume of 60 nl (SC), 150 nl (tegmentum), or 100 nl (V1) was injected at each site (SC: 3.75 mm caudal to Bregma, 0.6 mm lateral to midline, 1.3 mm ventral to Bregma; tegmentum: −4.3 mm caudal to Bregma, 1.5 mm lateral to midline, 3.2 and/or 3.4 mm ventral to Bregma; V1: 3.4 mm caudal to Bregma, 2.4 mm lateral to midline, 1.15 mm ventral to Bregma), at a rate of 20 nl/min. After removal of the needle, the scalp skin was sealed with tissue adhesive (n-butyl cyanoacrylate), lidocaine was applied to the wound, and the animals were placed on a heating pad until mobile. After surgery, animals were carefully monitored for proper wound healing, and injectable meloxicam (1–2 mg/kg) was administered for 48 h.

For BDA injections, P30-P40 mice were prepared for cranial surgery as described above and a glass pipette (20–40 μm tip diameter) containing a 5% solution of BDA (Invitrogen) in saline was lowered into the SC (from bregma: 3.8 mm posterior, 0.6 mm lateral, and 1.2 mm ventral), and BDA was iontophoretically ejected using 3 μA continuous positive current for 20 min. Post-operative care was carried out in the same manner as described for virus injections.

To express cre-recombinase in SC-PBG projection neurons that receive input from the retina, C57Blk6 pups (p15-18) received bilateral intravitreal injections of pAAV1.hSyn.Cre.WPRE.hGH. Each pup was anesthetized with isoflurane via a small nose cone, the sclera was pierced with a sharp-tipped glass pipette, and excess vitreous was drained. Another pipette filled with the AAV solution was inserted into the hole made by the first pipette. The pipette containing the AAV was attached to a picospritzer and a volume of approximately 1 μl of solution was injected into the eye. The nose cone used to administer isoflurane was then removed and, once alert, the pup was returned to the cage containing the dam and littermates.

### Histology for examination of the PBG and SC-PBG projections

Ten to fourteen days following the injection of viruses or 1 week after BDA injections, mice were deeply anesthetized with Avertin (0.5 mg/gm) or ketamine (100–150 mg/kg) and transcardially perfused with a fixative solution of 4% paraformaldehyde, or 2% paraformaldehyde and 2% glutaraldehyde in 0.1M phosphate buffer (PB). Additional GAD67-GFP mice that were not injected were also perfused for immunocytochemistry. In each case, the brain was removed from the skull and 70 μm thick coronal sections were cut using a vibratome (Leica Microsystems, Buffalo Grove, IL, USA). For BDA experiments, sections were incubated in avidin and biotinylated horseradish peroxidase (ABC solution, Vector Laboratories) overnight, reacted with nickel-enhanced diaminobenzidine (DAB) and processed for electron microscopy (described below). For the transsynaptic viral tracing experiments, selected sections were incubated overnight in a 0.1 μg/ml concentration of a rabbit polyclonal anti-GFP antibody (Millipore, Billerica, MA, USA, catalog #AB3080, RRID:AB_91337, created with highly purified native GFP from Aequorea victoria as an immunogen). Sections were then incubated in a 1:100 dilution of a biotinylated goat-anti-rabbit antibody (Vector Laboratories, Burlingame, CA, USA, catalog #BA-100, RRID:AB_23136061, 1 h), followed by ABC solution, (1 h), reacted with nickel-enhanced DAB and processed for electron microscopy (described below). All GFP antibody binding was confined to cells and terminals that contained GFP, as determined by their fluorescence under blue epifluorescent illumination; no staining was detected in sections that did not contain GFP. To identify axon terminals in the PBG positive for the vesicular GABA transporter (VGAT), selected tissue sections from GAD67 mice were incubated overnight in a 1:500 dilution of polyclonal anti-VGAT antibody (Synaptic Systems, Göttingen, Germany, catalog #131 103, RRID:AB_887870, created using synthetic peptide corresponding to residues near the amino terminus of rat VGAT). Sections were then incubated in a 1:100 dilution of goat-anti-rabbit antibody directly conjugated to a fluorescent compound (Alexa fluor 546, Cat. No. A11010, RRID:AB_2534077, Invitrogen, Carlsbad, CA, USA), washed in PB and mounted on slides to view using a confocal microscope.

### Slice preparation and optogenetic stimulation

Eight to twelve days following SC or tegmentum virus injections, mice were deeply anesthetized with isoflurane. Mice used for slice preparation ranged in age from P29 to P45 (average age p32). Mice were decapitated and the brain was removed from the head, chilled in cold slicing solution (in mM: 2.5 KCl, 26 NaHCO3, 2.5 KCl, 1.25 NaH2PO4, 10 MgCl2, 2 CaCl2, 234 sucrose, and 11 glucose) for 2 min, and quickly transferred into a Petri dish with room temperature slicing solution to block the brain for subsequent sectioning. Coronal slices (300 μm) were cut in cold slicing solution using a vibratome (Leica VT1000 S). Then slices were transferred into a room temperature incubation solution of oxygenated (95% O2/5% CO2) artificial cerebrospinal fluid containing the following (in mM: 126 NaCl, 26 NaHCO3, 2.5 KCl, 1.25 NaH2PO4, 2 MgCl2, 2 CaCl2, and 10 glucose) for 30 min to 6 h. Individual slices were transferred into a recording chamber, which was maintained at 32°C by an inline heater and continuously perfused with room temperature oxygenated ACSF (2.5 ml/min, 95% O2/5% CO2). Slices were stabilized by a slice anchor or harp (Warner Instruments, Hamden, CT, United States, 64– 0252). Neurons were visualized on an upright microscope (Olympus, BX51WI) equipped with both differential interference contrast optics and filter sets to detect fluorescence in the sections using a 4 × or 60 × water-immersion objective (Olympus, Center Valley, PA, United States) and a CCD camera. Recording electrodes were pulled from borosilicate glass capillaries (World Precision Instruments, Sarasota, FL, United States) by using a Model P-97 puller (Sutter Instruments, Novato, CA, United States). To record inhibitory post-synaptic currents (IPSCs) as well as excitatory post-synaptic currents (EPSCs) in PBG neurons, voltage-clamp recordings were conducted using a cesium-based internal solution containing (in mM): 117 Cs-gluconate, 11 CsCl, 1 MgCl2, 1 CaCl2, 0.1 EGTA, 10 HEPES, 2 Na2-ATP, 0.4 Na2-GTP, with pH adjusted to 7.3 using CsOH and osmolarity of 290 –295 mOsm. For current-clamp recordings, electrodes were filled with an intracellular solution containing the following (in mM): 117 K-gluconate, 13.0 KCl, 1 MgCl2, 0.07 CaCl2, 0.1 EGTA, 10 HEPES, 2 Na2-ATP, and 0.4 Na2-GTP, with pH adjusted to 7.3 using KOH and osmolarity 290 –295 mOsm. Biocytin (0.5%) was added to intracellular solutions to allow morphological reconstruction of the recorded neurons.

Whole-cell recordings were obtained from all regions of the PBG in both GAD2-Cre and PV-Cre mice, regardless of the AAV used (cre-dependent or non-cre-dependent). Recordings were obtained with an AxoClamp 2B amplifier (Molecular Devices), and a Digidata 1440A was used to acquire electrophysiological signals. The stimulation trigger was controlled by Clampex 10.3 software (Molecular Devices). The signals were sampled at 20 kHz, and data were analyzed offline using pClamp 10.0 software (Molecular Devices). For current-clamp recordings, voltage signals were obtained from cells with resting potentials of −50 to −65 mV. For voltage-clamp recordings, currents were recorded at 0 mV or −60 mV.

For photoactivation of SC-PBG or tegmentum-PBG terminals, light from a blue light-emitting diode (Prizmatix UHP 460) was reflected into a 60 × water-immersion objective. This produced a spot of blue light onto the submerged slice with a diameter of ∼0.3 mm. Pulse duration and frequency were under computer control. For repetitive stimulation, pulse duration was 1 ms. Synaptic responses were recorded using light intensities of 10–112 mW/mm^2^ (the intensity was measured using a light meter placed under the dry objective), and light pulse frequencies of 1, 2, 5, 10, and 20 Hz. To block GABAergic transmission pharmacologically, GABA receptors (GABA_*A*_) were blocked via bath application of the antagonist 2-(3-carboxypropyl)-3-amino-6-(4-methoxyphenyl)-pyridazinium bromide (SR95531, 20 μM; Tocris Bioscience, catalog #1262). To block NMDA and AMPA signaling pharmacologically, APV (10 μM; Sigma, catalog #A-5282) and/or CNQX (8 μM; Tocris Bioscience, catalog #0190)/DNQX (80 μM; Sigma, catalog #D0540-50MG) were bath applicated to block NMDA and AMPA receptors, respectively.

### Electron microscopy

Parabigeminal nucleus sections that contained DAB-labeled BDA or GFP were postfixed in 2% osmium tetroxide, dehydrated in an ethyl alcohol series, and flat embedded in Durcupan resin between two sheets of Aclar plastic (Ladd Research, Williston, VT, USA). Durcupan–embedded sections were first examined with a light microscope to select areas for electron microscopic analysis. Selected areas were mounted on blocks, ultrathin sections (70–80 nm, silver-gray interference color) were cut using a diamond knife, and sections were collected on Formvar-coated nickel slot grids. Selected sections were stained for the presence of GABA. A postembedding immunocytochemical protocol described previously (Bickford et al., 2010; Zhou et al., 2018; Masterson et al., 2019) was employed. Briefly, we used a 0.25 μg/ml concentration of a rabbit polyclonal antibody against GABA (Sigma-Aldrich, St. Louis, MO, USA, catalog #A2052, RRID:AB_477652). The GABA antibody was tagged with a goat-anti-rabbit antibody conjugated to 15-nm gold particles (BBI Solutions USA, Madison, WI, USA, catalog# GAR12/0.25, RRID:AB 1769132). The sections were air dried and stained with a 10% solution of uranyl acetate in methanol for 30 min before examination with an electron microscope.

### Processing of cells filled during physiological recording

Following recording, slices were placed in a fixative solution of 4% paraformaldehyde in PB for at least 24 h. The sections were then rinsed in PB and incubated overnight in a 1:1000 dilution of streptavidin conjugated to AlexaFluor-633 (Invitrogen) in PB containing 1% Triton X-100. The following day, the slices were washed in PB, preincubated in 10% normal goat serum in PB, and then incubated overnight in a 1:500 dilution of a rabbit anti-DSred antibody (Clontech, catalog #632496) in PB with 1% normal goat serum. The following day, the sections were rinsed in PB and incubated for 1 h in a 1:100 dilution of a goat-anti-rabbit antibody conjugated to AlexaFluor-546 (Invitrogen). The sections were then rinsed in PB and mounted on slides to be imaged with a confocal microscope (Olympus FV1200BX61).

### Morphological analysis of PBG neurons

To classify neurons recorded *in vitro* by their morphological characteristics, confocal-imaged, biocytin-filled cells were first traced in PowerPoint by hand using the marker tool. Images of the resultant 2-D traces were then uploaded in ImageJ (Fiji) where they were first binarized and then analyzed via the “Sholl Ring” analysis tool. The “start radius” for these analyses was set to 15 pixels, with a “step size” (Sholl ring size) of 10 pixels. The center and end radius of the Sholl rings for each cell were defined by a region of interest (ROI, straight line) drawn from the approximate center of the soma to the most distal point in the dendritic arbor before performing the analysis. The Sholl ring diagrams produced by these ImageJ analyses were then used to quantify the number of dendritic “crossings” in each of 8 radially organized sections around the center (defined by the ROI described above) of each cell. Cells in which less than 20% of these crossings could be found in 4 contiguous radial sections in any orientation were considered “Asymmetric.” Cells where any 4 contiguous sections always represented 20% or more of the total crossings regardless of orientation were considered “Stellate.” Cells in which more than 50% of total crossings could be found in 2 symmetrical (on opposite sides) sections were classified as “Narrow-Field.” These Narrow-Field cells could be further classified as symmetric or asymmetric based on the criteria described above for the Asymmetric and Stellate (symmetric) cell types. Radar plots illustrating the orientation and density of cell arbors based on the number of Sholl ring crossings in each 1/8 radial section were made using Microsoft Excel. Radial sections were defined via a lined overlay that split each cell’s anatomy into 8 sections of equal radial size, centered on each cell’s soma (also the origin of each ROI used to define the parameters of the Sholl rings).

### Electron microscopy image processing and analysis

To quantify the number of glutamatergic or GABAergic terminals in the PBG’s “core” versus its “shell” based on the tissue’s ultrastructure, 7 × 7 (∼2,000 μm^2^) montages of ultrathin sections, with each image taken at a magnification of 10,000× (∼7.8 × 5.8 μm), were collected on the electron microscope. These montages were then “stitched” together using the image processing software, 3mod. The montages were then pieced together by hand using Microsoft PowerPoint to create a “Montage Collage” for each ultrathin section of interest. To define the “core” and “shell” of the PBG for each collage, an ellipse was drawn around the approximate edge of the outermost cell soma of the core, with a second ellipse drawn around the first with a radius exactly 4 microns larger. This second, larger ellipse defined the bounds of the PBG core for each ultrathin section of interest. The area outside this ellipse containing SC-PBG terminals was defined as the “shell.” Terminals identified in each collage were marked for quantification using the “sculpt” tool in 3mod, which tracks the number of contours (points in this case) used to create an area of interest. After marking each collage in this way to identify all glutamatergic and GABAergic (labeled with antibodies conjugated to gold particles) terminals, the total number of each was quantified after the PBG core and shell had been spatially defined as described above.

## Results

### Topography of SC-PBG projections

**FIGURE 1:**
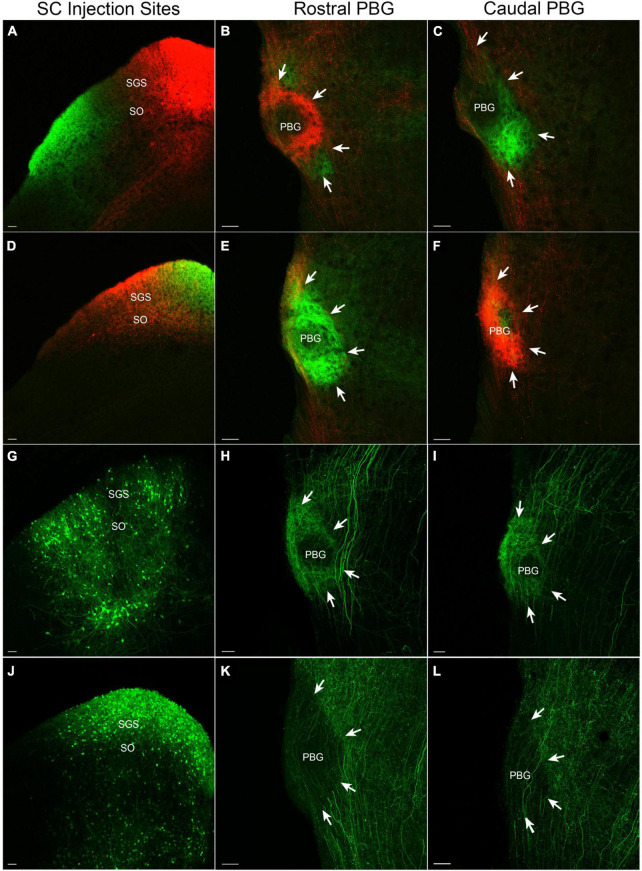
SC-PBG projection patterns. The left column illustrates SC injection sites that labeled axons in the rostral (middle column) and caudal (right column) PBG (perimeter indicated by white arrows). All sections are shown in the coronal plane. **(A)** An AAV that expresses tdTomato injected in the medial SC labels projections primarily to the rostral PBG **(B)**, while an AAV injection that expresses GFP in the lateral SC labels projections primarily to the caudal PBG **(C)**. **(D)** An AAV targeted to the caudal SC that expresses tdTomato primarily labels projections to the caudal PBG **(F)**, while an AAV injected into the rostral SC that expresses GFP mostly labels projections to the rostral PBG **(E)**. **(G)** A cre-dependent AAV that expresses GFP injected into the SC of a PV-cre mouse labels a dense population of PV cells and their projections to the rostral and caudal PBG [**(H,I)**, respectively]. **(J)** A cre-dependent AAV that expresses GFP injected into the SC of a GAD2-cre mouse labels a dense population of GABAergic neurons in the SC and their projections to both the rostral and caudal PBG [**(K,L)**, respectively]. SGS, stratum griseum superficiale; SO, stratum opticum. Scale bars = 50 μm in all panels.

The injection of two different viruses in different regions of the same SC revealed a coarse topography of SC-PBG projections. For example, injections that induced cells in the medial SC to express TdTomato and cells in the lateral SC to express GFP (Figure 1A) labeled projections to the ipsilateral PBG that were largely non-overlapping. Injections in the medial SC labeled axons and terminals in the more rostral regions of the PBG (Figure 1B), whereas injections in the lateral SC labeled axons and terminals in more caudal regions of the PBG (Figure 1C). Likewise, injections in the rostral and caudal regions of the same SC labeled non-overlapping projections to the ipsilateral PBG. Injections in the caudal SC (Figure 1D) primarily labeled projections to the caudal PBG (Figure 1F) while injections in the rostral SC labeled projections that primarily targeted the more rostral regions of the PBG (Figure 1E). Thus, the SC projections to the mouse PBG roughly preserve the retinotopic organization of the superficial layers of the SC; the rostral PBG likely receives input from the upper/nasal visual field (represented in the rostral and medial regions of the SC) while the caudal PBG likely receives input from lower/peripheral visual field (represented by the caudal and lateral regions of the SC).

### SC projections define “core” and “shell” regions of the PBG

The SC virus injections revealed another organizational feature of the PBG which we define as the “core” (center of the PBG) and “shell” (periphery of the PBG). In fluorescent images, projections from the SC appear to be more densely distributed in the shell of the PBG (particularly apparent in the rostral PBG; Figure 1 panels B and E). As described below, our ultrastructural studies revealed that the core contains densely packed somata, while the shell contains more myelinated axons. Thus, the density of SC projections in the shell detected via fluorescence likely represent the abundance of myelinated axons traveling from the SC to innervate the PBG.

### GABAergic and parvalbumin-expressing SC-PBG projections

The SC neurons that project to the PBG include at least two distinct populations: neurons that express parvalbumin (Shang et al., 2015, 2018) and GABAergic neurons that do not express parvalbumin (Whyland et al., 2020). These two PBG-projecting populations represent only a fraction of all the GABAergic and/or parvalbumin-expressing neurons within the SC (Villalobos et al., 2018; Whyland et al., 2020). Moreover, these two PBG-projecting populations may be further subdivided, and/or additional populations of SC neurons may project to the PBG (Gale and Murphy, 2014, 2018; Hoy et al., 2019). However, we focused on these two broad categories for an initial evaluation of SC-PBG circuits.

Viruses were injected in the SC of PV-cre (Figure 1G) or GAD2-cre (Figure 1J) mice to induce the expression of GFP in a cre-dependent manner. As previously described (Shang et al., 2015), parvalbumin-expressing neurons densely innervate both the rostral (Figure 1H) and caudal PBG (Figure 1I). GABAergic SC neurons also innervate the rostral (Figure 1K) and caudal PBG (Figure 1L). However, GABAergic projections from the SC to the PBG were not as dense as those arising from parvalbumin-expressing neurons in the SC. GABAergic projections from the SC were also densely distributed in regions of the tegmentum surrounding the PBG, whereas projections from parvalbumin SC neurons to the tegmentum surrounding the PBG were sparse.

### Ultrastructure and GABA content of SC-PBG terminals

**FIGURE 2:**
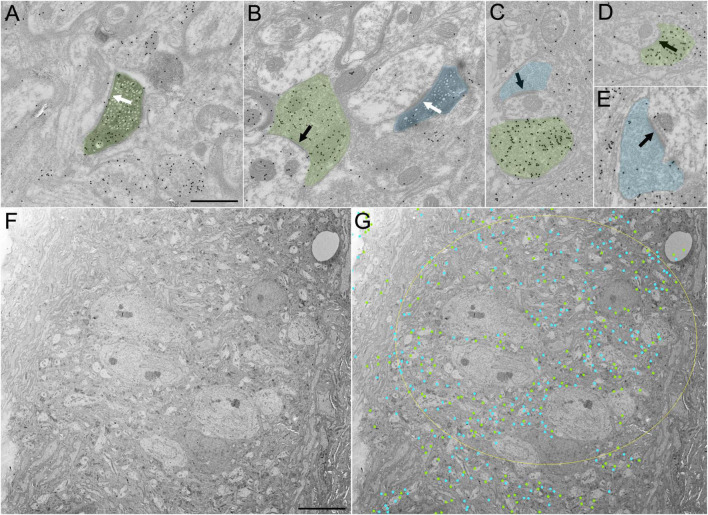
Ultrastructure and distribution of GABAergic and non-GABAergic terminals in the “core” and “shell” regions of the PBG. **(A)** A GABAergic (high density of overlying gold particles) BDA-labeled (dark reaction product) synaptic terminal (green) originating from the SC synapses (white arrow) with a non-GABAergic dendrite (low density of overlying gold particles) in the PBG. **(B)** A non-GABAergic BDA-labeled terminal originating from the SC (blue) synapses (white arrow) on a non-GABAergic dendrite in the PBG. A nearby GABAergic (green) terminal synapses with a non-GABAergic dendrite (black arrow). **(C–E)** Examples of GABAergic (green) and non-GABAergic (blue) terminals in the PBG. Synapses indicated with black arrows. **(F)** Low magnification image of the PBG “core” in an ultrathin section that was analyzed for GABAergic and non-GABAergic axon terminal density. **(G)** Same image as in panel **(F)** but with labeling showing the approximate location of identified GABAergic terminals (green dots) and non-GABAergic terminals (blue dots). The yellow ellipse denotes the approximate boundary of the PBG “core”. Scale bars in panels **(A–E)** = 600 nm, **(F,G)** = 8 μm.

To examine the ultrastructure and distribution of GABAergic and non-GABAergic tectal terminals in the PBG, we injected the SC of C57Blk6 mice with BDA to label SC-PBG terminals and additionally stained sections containing labeled terminals with an antibody against GABA tagged with gold particles. SC-PBG terminals were identified by the DAB reaction product contained within them (Figures 2A, B) and were separated into GABAergic (green overlay, Figures 2A–D) and non-GABAergic (blue overlay, Figures 2B, C, E) categories based on a qualitative assessment of the density of overlying gold particles. We examined a total of 103 SC-PBG terminals that were engaged in synapses. Of these, only 7 (∼7%) were GABAergic, corroborating the differences in the density of SC projections labeled in PV-cre and GAD-cre mice (Figures 1G–L). The PBG dendrites postsynaptic to both GABAergic and non-GABAergic SC terminals were found to be non-GABAergic (i.e., contained a low density of gold particles; Figures 2A, B).

To determine whether GABAergic and non-GABAergic SC-PBG populations are differentially innervated by retinal ganglion cells or V1, we placed injections of a virus that is transported transynaptically to expresses cre-recombinase in postsynaptic cells in the eyes or V1 of C57Blk6 mice; we followed these injections with cre-dependent virus injections in the SC to label SC-PBG projection populations with GFP in an input-defined manner. We then used an antibody against GFP to label the terminals in the PBG with a DAB reaction product and prepared the tissue for electron microscopy. As for the BDA-labeled terminals described above, we additionally stained these terminals with an antibody against GABA tagged with gold particles so that we could examine the distribution of GABAergic and non-GABAergic synaptic terminals in the PBG. These experiments did not reveal significant differences in the proportions of GABAergic and non-GABAergic SC-PBG projections arising from neurons that receive input from the retina or V1. For both labeled populations (postsynaptic to retina or V1 inputs) a modest proportion of labeled terminals in the PBG contained GABA. For SC-PBG projections arising from neurons that receive retinal input, 9.27% (28 of 302 synaptic terminals) contained GABA. For SC-PBG projections arising from neurons that receive V1 input, 4.8% (11 of 229 synaptic terminals) contained GABA. These proportions were not significantly different from those revealed in our BDA-injection experiments (7%; *p* = 0.22004, *p* = 0.77087, respectively; two proportion z-test). The sizes of GABAergic (0.586 μm^2^ ± 0.403, *n* = 39) and non-GABAergic (0.600 μm^2^ ± 0.456, *n* = 492) SC-PBG synaptic terminals were also not found to be significantly different (Mann–Whitney, *p* = 0.7957).

### GABAergic and non-GABAergic synaptic terminal density in the PBG “core” and “shell”

We next examined the overall distribution of GABAergic and non-GABAergic terminals in the PBG, comparing “core” and “shell” regions. As illustrated in Figures 2F, G, the core contains densely packed neuron somata, while the shell contains a dense distribution of myelinated axons. A total of 1,405 terminal profiles (that contained presynaptic vesicles) were identified in 2 montages of sections that contained the core and shell. The sections were stained with the GABA antibody so that the proportions of GABAergic and non-GABAergic terminals could be quantified. In each montage, a large portion of the terminals contained GABA (montage 1: 44% of terminals in the core and 42% in the shell were GABAergic; montage 2: 49.8% of terminals in the core and 40.9% in the shell were GABAergic). The proportions of GABAergic terminals in the core versus shell were not found to be significantly different (*p* = 0.98324, two proportion z-test) but both were significantly higher than the proportion of SC-PBG terminals that contained GABA [*p* < 0.001 (3.71739 × 10^–60^), two proportion z test]. The density of terminal profiles was also similar in the core and shell of the PBG (montage 1: core contained 266 terminals identified within 1,693 μm^2^ or 0.157 terminals/μm^2^, shell contained 336 terminals identified within approximately 2,840 μm^2^ or 0.118 terminals/μm^2^; montage 2: core contained 297 terminals within 2,597 μm^2^ or 0.114 terminals/μm^2^, shell contained 506 terminals within approximately 4,063 μm^2^ or 0.125 terminals/μm^2^). The terminal densities were not found be significantly different in the core versus the shell (*p* = 0.924712, two proportion z test).

### Non-tectal GABAergic terminals in the PBG are labeled in the GAD67-GFP line

**FIGURE 3:**
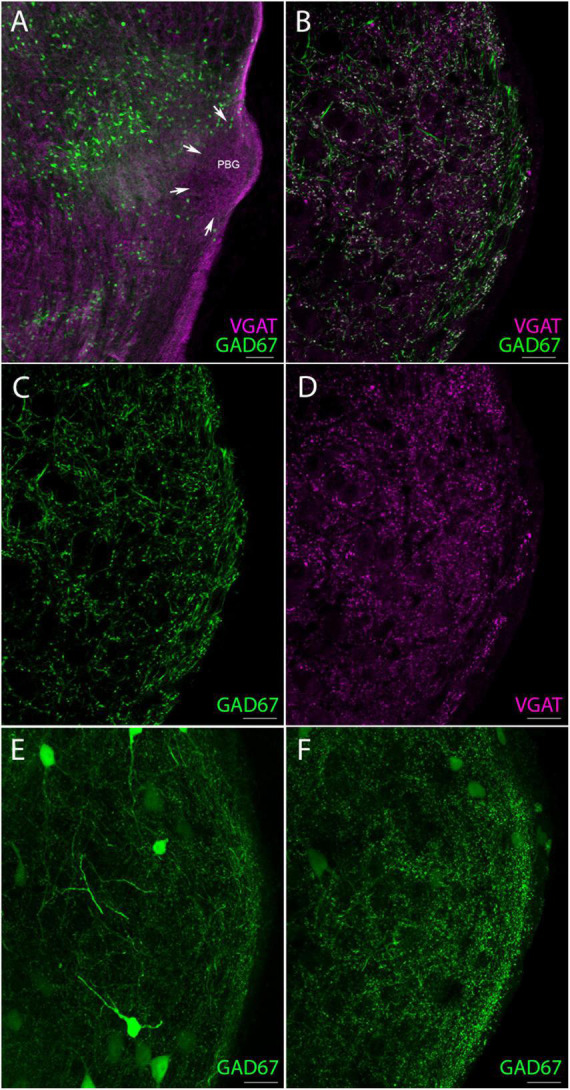
GAD67 neurons provide a source of non-tectal GABAergic terminals in the PBG. **(A)** Low magnification confocal image of numerous GAD67 neurons (green) fluorescently labeled with GFP in the GAD67-GFP reporter mouse, occupying areas of the brainstem just medial to the PBG (perimeter denoted by white arrows) which is also labeled with an antibody against the vesicular GABA transporter (VGAT, magenta). **(B)** High magnification confocal image of GAD67 (green) and VGAT + (magenta) fibers intermingled in the PBG, with many fibers clearly co-labeled (white). **(C)** Same image as in panel **(B)** but with only GAD67 fibers shown. **(D)** Same image as in panel **(B)** but with only antibody labeled VGAT + fibers shown. **(E,F)** Two other confocal images of the PBG from a GAD67-GFP mouse. GFP-labeled fibers are seen throughout the nucleus as well as a few GAD67 + somata. Scale bar in panel **(A)** = 100 μm, **(B–F)** = 20 μm.

Since we found that less than 10% of the labeled SC terminals in the PBG contain GABA, while our montage analysis revealed that over 40% of the terminals in the PBG contain GABA, the PBG must be innervated by other sources of GABAergic neurons. In fact, when we examined confocal images of the PBG in GAD67-GFP mice we observed an abundance of GFP-labeled neurons in the brainstem adjacent to the PBG (Figure 3A) and occasional GFP-labeled neurons within the PBG (Figures 3E, F). Additionally, in GAD67-GFP mice the PBG contains a dense population of GFP-labeled fibers that can also be labeled with antibodies against vesicular GABA transporter (vGAT; Figures 3B–D), a protein that is contained within synaptic terminals (Chaudhry et al., 1998). In a previous study, we found that GAD67-GFP neurons in the SC do not project to the PBG (Whyland et al., 2020). Therefore, the PBG terminals that contain GFP in the GAD67-GFP line identify an additional, non-tectal source of GABAergic input to the PBG. These may arise from the GFP-labeled neurons in the tegmentum surrounding the PBG and/or the sparse GFP-labeled neurons in the PBG.

### Optogenetic activation of tegmentum-PBG terminals in GAD2-cre mice

**FIGURE 4:**
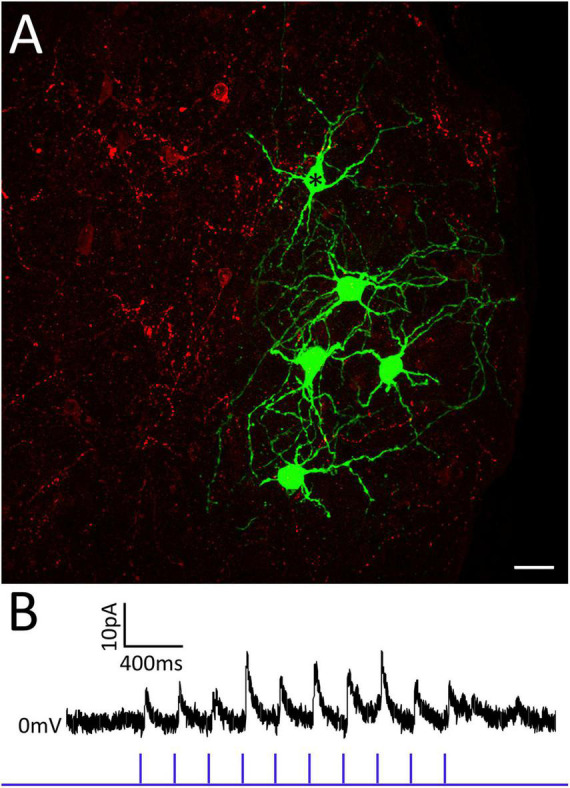
The tegmentum surrounding the PBG provides sparse GABAergic input to the PBG. Panel **(A)** illustrates 5 PBG neurons (green) that were filled with biocytin during recording and optogenetic activation of GABAergic axons and terminals originating from the surrounding tegmentum (red). Only one of these neurons (asterisk) responded with inhibitory postsynaptic currents **(B)** during photoactivation of GABAergic tegmental terminals. PBG neurons recorded in other slices were also non-responsive to tegmental input.

To determine if the tegmentum surrounding the PBG is a source of GABAergic input to PBG neurons, a virus that induces the expression of the light-sensitive cation channel, channelrhodopsin (ChR2) in the presence of cre-recombinase was injected just medial to the PBG in GAD2-cre mice. Two weeks later, slices of the PBG from injected mice were prepared for *in vitro* recordings. Whole-cell patch clamp recordings of PBG neurons were obtained and GABAergic axons and terminals that expressed ChR2 were activated via 1 ms pulses of blue LED light (1, 2, 5, 10, 20 Hz). The recording pipettes contained biocytin so that we could subsequently identify the recorded neurons (Figure 4A, green). Activation of tegmental GABAergic axons and terminals induced few postsynaptic responses in the PBG. Of eleven neurons recorded in slices that contained labeled terminals in the PBG (Figure 4A, red), only one neuron responded with inhibitory postsynaptic currents (Figure 4B). Moreover, this responsive neuron (Figure 4A, asterisk) was surrounded by four non-responsive neurons. Therefore, the tegmentum provides a sparse source of GABAergic input to the PBG. Additional non-tectal sources likely contribute to the dense distribution of GABAergic terminals within the PBG identified by our electron microscopic analyses.

### Optogenetic activation of SC-PBG terminals in GAD2-cre and PV-cre mice

**FIGURE 5:**
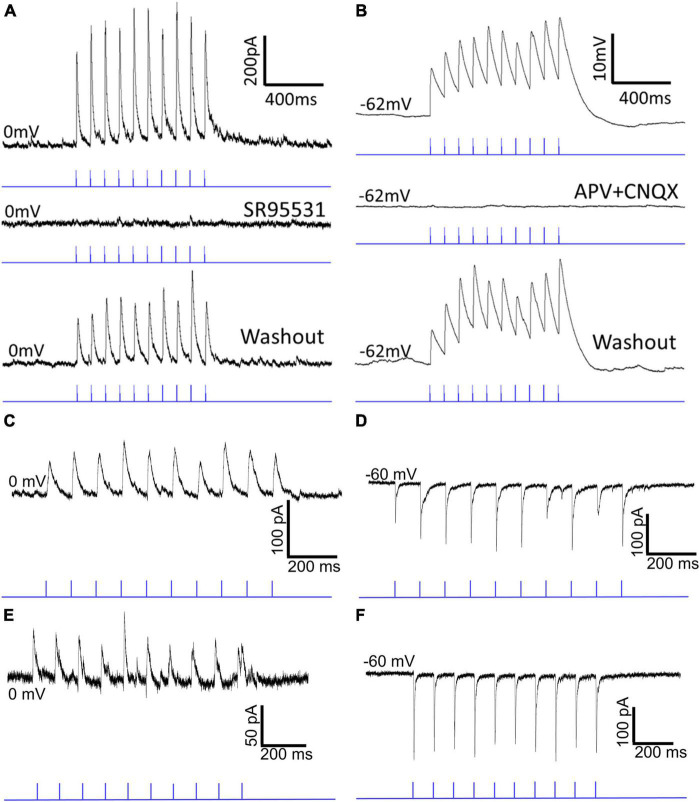
Optogenetic activation of GABAergic and glutamatergic SC-PBG terminals in GAD2-cre and PV-cre mice. Panel **(A)** shows a current trace from an *in vitro* whole-cell patch clamp experiment in which a cre-dependent AAV that expresses the light-sensitive cation channel, Channelrhodopsin, was injected into the SC of a GAD2-cre mouse. Optical stimulation of axon terminals in the tissue slice with 1 ms pulses of blue light reliably produces IPSCs (top trace) in the patched neuron that can be extinguished [middle trace of panel **(A)**] via the administration of the selective GABA-A antagonist SR95531 into the circulating ACSF, confirming the presence of GABAergic inputs to the PBG. After washing out this antagonist, the same cell resumes IPSC responses to optical stimulation [bottom trace of panel **(A)**]. Panel **(B)** shows a voltage trace from a similar experiment in which a cre-dependent AAV was injected into the SC of a PV-cre mouse. Optical stimulation of axon terminals in the tissue slice reliably evoked EPSPs that could be silenced with the administration of the NMDA and AMPA blockers, APV and CNQX, confirming that PV + SC-PBG projections are glutamatergic. Washing out these antagonists recovered the excitatory responses seen in the first trace of panel **(B)** [bottom trace of panel **(B)**]. **(C)** An example current trace demonstrates patch-clamp experiments in which a non-cre-dependent AAV was injected into the SC (thus infecting all neuronal cell-types). Both IPSCs **(C)** and EPSCs **(D)** can be reliably evoked depending on whether the cell is held at 0 mV **(C)** or –60 mV **(D)**. **(E,F)** Voltage traces from another example recorded neuron in which both IPSCs and EPSCs could be reliably evoked via optical stimulation of axon terminals originating from the SC.

To examine the postsynaptic effects of activation of GAD2 and PV SC-PBG terminals, a virus that induces the expression of the light-sensitive cation channel, channelrhodopsin (ChR2) in the presence of cre-recombinase was injected into the SC of GAD2-cre and PV-cre mice. Two weeks later, slices of the PBG of injected mice were prepared for *in vitro* recordings. Whole-cell patch clamp recordings of PBG neurons were obtained and SC-PBG axons and terminals that expressed ChR2 were activated via 1 ms pulses of blue LED light (1, 2, 5, 10, 20 Hz). Examples of the recordings are illustrated in Figure 5 and the results are summarized in Tables 1, 2. In slices from injected GAD2-cre mice, photoactivation of SC terminals consistently elicited inhibitory postsynaptic currents (IPSCs) greater than 200 pA in PBG neurons held at 0 mV during voltage clamp recordings (Figure 5A). Adding the GABAergic receptor antagonist SR95531 to the circulating ACSF solution extinguished these IPSCs (second trace in panel 5A), confirming that the presynaptic release of GABA was responsible for the observed IPSCs. Wash out of the antagonist from the ACSF solution restored the evoked IPSCs (bottom trace in panel 5A). In contrast, in slices from injected PV-cre mice, photoactivation of SC terminals during current clamp recordings from PBG neurons (resting membrane potentials typically ∼−60 mV) consistently elicited excitatory postsynaptic potentials (EPSPs) with amplitudes of 10mV or more (evoked EPSPs triggered action potentials in 5 of the recorded neurons). Adding the NMDA receptor antagonist APV and the AMPA receptor antagonist CNQX abolished the EPSPs (second trace, panel 5B). Wash out of these glutamatergic receptor antagonists restored the evoked EPSPs (bottom trace, panel 5B). In summary, these experiments confirmed that GAD2 SC neurons release GABA in the PBG, PV SC neurons release glutamate in the PBG, and these two inputs elicit opposing effects on their postsynaptic targets.

**TABLE 1:** GABAergic and glutamatergic responses to SC input and PBG morphology.

Responsive cells *N* = 40	Max amplitude @ 10 Hz	Membrane resistance	Filled cells (*n*)	Stellate	Asymmetric	Narrow field
Cre-dependent AAV in SC of PV-Cre, current clamp recordings, *n* = 13	15.11 mV (± 6.6)	302 MΩ (± 139)	(8/13)	(2/8)	(4/8)	(2/8)
Non-cre-dependent AAV in SC, current clamp recordings, *n* = 20	11.77 mV (± 7.06)	663 MΩ (± 199)	(11/20)	(5/11)	(4/11)	(2/11)
All current clamp recordings *n* = 33	13.09 mV (± 6.97)	521 MΩ (± 250)	(19/33)	(7/19)	(8/19)	(4/19)
Cre-dependent AAV in SC of GAD2-cre, voltage clamp recordings, *n* = 7	274.5 pA (± 209)	–	(0/7)	(0/7)	(0/7)	(0/7)
Non-responsive filled cells current clamp *n* = 26	–	542 ± 180 MΩ	26	20/26	4/26	2/26
Non-responsive filled cells voltage clamp *n* = 6	–	–	6	3/6	1/6	2/6

**TABLE 2:** GABAergic and glutamatergic SC input convergence and PBG morphology.

Non-cre-dependent AAV in SC, voltage clamp recordings	Max amplitude EPSC (−60 mV, 10 Hz)	Max amplitude IPSC (0 mV, 10 Hz)	EPSC/IPSC ratio (max amplitudes @ 10 Hz)	Mean paired pulse ratio @ −60 mV (10 Hz)	Mean paired pulse ratio @ 0 mV (10 Hz)
All responsive *n* = 26	251.2 pA (±235.8)	174.1 pA (±113.2)	1.74 (±1.29)	1.206 (±0.481)	1.011 (±0.191)
Stellate *n* = 8	218.8 pA (± 138.7)	142.3 pA (± 45.6)	1.71 (± 1.12)	1.193 (± 0.413)	1.017 (± 0.167)
Asymmetric *n* = 5	298.8 pA (±213.5)	180.3 pA (±54.2)	1.97 (±1.45)	1.262 (±0.313)	0.964 (±0.07)
Narrow-field *n* = 3	457.2 pA (±338.3)	166.7 pA (±51.4)	2.91 (±1.08)	1.567 (±0.847)	0.897 (±0.143)
Unfilled *n* = 7	180.1 pA (±190.3)	200 pA (±178.5)	0.99 (±0.87)	1.0 (±0.233)	1.088 (±0.246)
Other statistics	Fired only action potentials at −60 mV *n* = 3	Voltage clamp excitatory responses only *n* = 4	Voltage clamp inhibitory responses only *n* = 2	Voltage clamp excitatory response at 0 and −60 mV *n* = 2	Excitatory and inhibitory responses *n* = 18

### GABAergic and glutamatergic SC inputs convergence on single PBG neurons

Next, we tested whether these GABAergic and glutamatergic SC inputs synapse on separate PBG populations or whether they converge to innervate single PBG neurons. For these experiments, we injected a virus in the SC to express ChR2 in a non-cre-dependent manner. Then in slices from injected mice we photoactivated SC terminals while recording from PBG neurons voltage clamped at 0 mV or −60 mV. These experiments revealed that the majority of responsive PBG neurons (18/26, 69.2% of responsive cells) responded with both IPSCs and EPSCs. Example traces are illustrated in Figure 5 and the results summarized in Table 2. In the example trace shown in Figure 5C, 1 ms blue light pulses evoked IPSCs of ∼50 pA while the cell was held at 0 mV. When the same cell was held at −60 mV, blue light pulses evoked EPSCs of ∼100 pA (Figure 5D). Another example of these mixed responses recorded from the same PBG neuron is shown in panels 5E and 5F. It should be noted that the amplitudes of evoked EPSCs were often larger than their IPSC counterparts, and additionally that most cells that did not have mixed responses only responded with EPSCs (6/8, 75%). As summarized in Table 2, the average ratio of EPSC to IPSC maximum amplitudes recorded in single PBG neurons was 1.74 ± 1.29. This observation is congruent with virus labeling and electron microscopic results which suggest that GABAergic terminals make up a minority of the input from the SC to the PBG. Finally, as summarized in Table 2, both EPSC and IPSC amplitudes increased in amplitude during the course of 10 Hz photostimulation (a paired pulse ratio was quantified by dividing the average amplitude of the 2nd through 10th EPSC/IPSC by the amplitude of the first EPSC/IPSC in the train). For EPSCs, the mean paired pulse ratio @ 10 Hz was 1.206. For IPSCs, the mean paired pulse ratio @ 10 Hz was 1.011. These ratios were not found to be significantly different (Mann–Whitney, *p* = 0.3178).

### PBG cell morphology

**FIGURE 6:**
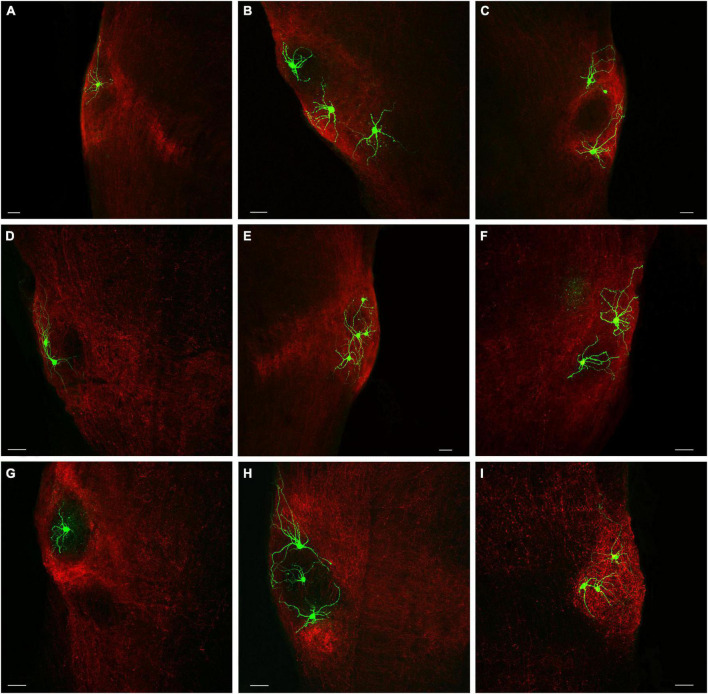
Biocytin-filled PBG cells exhibit a variety of neuronal morphologies. **(A–I)** The addition of biocytin to the internal recording solution in the whole-cell *in vitro* physiology experiments enabled the visualization of recorded cells (green). The recorded PBG cells are surrounded by terminals labeled via virus injections in the SC (red). Scale bars = 50 μm for all panels.

**FIGURE 7:**
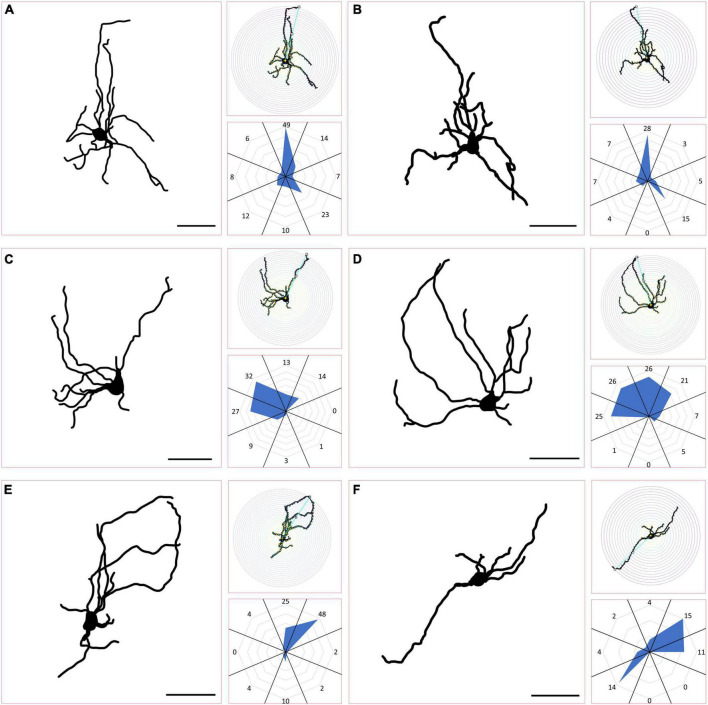
Classification of PBG morphological cell types. **(A,B)** Example traces from biocytin-filled PBG neurons classified as stellate can be seen in the large panels, accompanied to the right by their respective Scholl ring diagrams (top, small panels) and radar orientation plots (bottom, small panels), demonstrating how their morphological characteristics were quantified for each. Neurons where 4 contiguous radial sections always comprised 20% or more of the total Scholl ring crossings were considered stellate. **(C,D)** Example traces of PBG neurons classified as asymmetric. Asymmetric cells had 4 contiguous radial sections comprising less than 20% of the total Scholl ring crossings. **(E,F)** Example traces of PBG neurons classified as narrow field, with one example of an asymmetric narrow field cell **(E)** and one example of a symmetric narrow field cell **(F)**. Narrow field cells had two radial sections on opposite sides comprising more than 50% of the total Scholl ring crossings.

We next wanted to determine whether any of the morphological features of the PBG cells filled with biocytin during in our *in vitro* slice experiments correlated with recorded physiological characteristics. As illustrated in Figure 6, the biocytin-filled PBG neurons exhibited a variety of sizes and morphologies. As described in the methods section, Sholl ring analysis of dendritic arbors was used to categorize biocytin-filled PBG neurons as stellate, asymmetric, narrow-field symmetric, or narrow-field asymmetric. Examples of each cell type are shown in Figure 7. We were able to successfully fill 70 PBG neurons in our slice experiments. Whether these cells were responsive to optical stimulation or not, the majority of biocytin-filled cells were categorized as stellate (37 of 70 or 52.9%), with asymmetric cells forming the next largest group (21 of 70 or 30%). Narrow-field cells formed the smallest group (12/70, 17.1%), with the majority of these categorized as narrow field symmetric (8 of 12 or 66.6% symmetric, 4 of 12 or 33.3% asymmetric). However, with exception of soma size which had a modest inverse correlation to membrane resistance (measured in current clamp experiments, *n* = 23; correlation coefficient: −0.467; *p* = 0.0248), none of the other physiological characteristics that we measured exhibited a strong correlation with the morphologies analyzed (max amplitude of responses, EPSC/IPSC ratio, and paired-pulse ratios; Kruskal Wallis, *p* > 0.9999 for most comparisons). It should be noted that the coronal slices employed could have truncated some of the reconstructed neurons used for this morphological analysis, potentially altering their classification.

## Discussion

Our anatomical and physiological dissections of SC-PBG circuits in the mouse yielded the following conclusions: (1) SC-PBG projections exhibit a rough topography which may preserve the retinotopy of the SC. (2) The PBG can be divided into “core” regions that contain densely packed somata, and “shell” regions that contain more myelinated axons, but both regions contain similar densities of synaptic terminals. (3) GABAergic and non-GABAergic SC neurons that project to the PBG receive input from the retina and V1. (4) Less than 10% of SC projections to the PBG contain GABA. (5) Approximately 40% of the overall input to the PBG is GABAergic. (6) Terminals labeled in GAD67-GFP mice indicate the PBG receives GABAergic input from non-tectal sources, partially provided by the tegmentum surrounding the PBG. (7) GABAergic SC-PBG projections do not contain parvalbumin and inhibit PBG neurons. (8) SC-PBG projections that express parvalbumin release glutamate to excite PBG neurons. (9) Most PBG neurons receive convergent excitatory and inhibitory input from the SC. (10) PBG neurons exhibit a variety of dendritic morphologies, but these did not correlate with the physiological properties measured in the current study. These conclusions are discussed in further detail below.

### Topography and core/shell organization of the SC-PBG projections

Our virus injections indicate that the rostral/medial SC targets the rostral PBG while the caudal/lateral SC targets the caudal PBG. Thus, the rostral PBG of the mouse likely receives input from the upper/nasal (binocular) visual field while the caudal PBG likely receives input from lower/peripheral visual field. Retrograde tracing experiments in the common Degu, a diurnal rodent, show a similar pattern. In this species, parasagittal sections revealed that the PBG can be clearly divided into anterior and posterior subdivisions, each with distinct reciprocal connectivity with the SC; cells located in the rostral/medial SC tended to project to the rostral PBG, while cells located in the caudal/lateral SC projected to the caudal PBG (Deichler et al., 2020). Moreover, it was found that the rostral PBG projects to the rostral/medial regions of the contralateral SC, suggesting that the rostral subdivision of the PBG may be specialized for the detection of predators in the binocular visual field (Deichler et al., 2020; Tokuoka et al., 2020). In the cat, physiological recordings and retrograde tracing from the SC also indicate that the PBG is retinotopically organized and that the rostral PBG may be a specialized zone representing the binocular visual field (Sherk, 1978, 1979b).

Our virus labeling experiments also highlighted an intriguing pattern of innervation in the PBG in which the edges of the nucleus (“shell”) consistently appear more densely innervated by SC inputs. Our ultrastructural analysis indicates that this pattern may simply reflect the high density of myelinated axons in the periphery of the nucleus. Although the center (“core”) of the nucleus contains more somata, we found that the density of synaptic terminals in each region was similar. Therefore, SC axons may enter the PBG from the “shell,” but it does not appear to be more extensively innervated relative to the “core.” Since the “core” vs. “shell” pattern is most obvious in rostral sections of the PBG, it may be related to the rostral and caudal PBG subdivisions observed (Deichler et al., 2020) in the Degu. However, future studies utilizing parasagittal sections and/or retrograde labeling of PBG neurons that project to the SC will be necessary to fully explore this possibility.

### GABAergic and glutamatergic SC projections converge on single PBG neurons

Our *in vitro* physiology experiments confirmed the presence of two SC-PBG projection populations with opposing effects on the PBG: GABAergic neurons (that don’t contain parvalbumin) inhibit PBG neurons, and glutamatergic neurons (which include neurons that contain parvalbumin) excite PBG neurons. While our electron microscopy studies indicate that less than 10% of the SC input to the PBG is GABAergic, we found that the majority of PBG neuron receive both GABAergic and glutamatergic input from the SC. Thus, the GABAergic and glutamatergic SC-PBG pathways are not segregated but converge to influence the receptive field properties of most PBG neurons.

Transynaptic retrograde tracing studies have revealed that SC-PBG neurons cells receive input from retinal ganglion cells, V1, as well as other sources (Reinhard et al., 2019). Anterograde transsynaptic tracing studies suggested that V1 does not innervate GABAergic projection cells in the SC (Zingg et al., 2017). Thus, we reasoned that the use of transsynaptic viral tracing techniques to label SC neurons that receive input from V1 would not label any GABAergic synaptic terminals in the PBG. Instead, we found no differences in the proportion of GABAergic and non-GABAergic SC-PBG terminals following BDA injections in SC, or in experiments where we transynaptically labeled SC neurons that receive input from the retina or V1. Therefore, whether GABAergic and non-GABAergic SC-PBG neurons receive distinct presynaptic inputs remains an open question.

It is also unclear how many different types of SC-PBG neurons are included within these two broad categories of GABAergic and glutamatergic neurons. Our experiments indicate that glutamatergic SC cells that project to the PBG do include those that contain parvalbumin, but they may also include neurons that do not express parvalbumin. Furthermore, our previous experiments indicate that GABAergic SC neurons that project to the PBG can exhibit stellate, NFV or horizontal morphologies (Whyland et al., 2020). Therefore, the GABAergic and glutamatergic SC-PBG populations likely include subcategories with distinct morphologies and physiological properties, all contributing to the complex receptive field properties of PBG neurons.

### Morphology of PBG neurons

The mouse PBG is approximately ∼250–300 microns in its anterior-posterior extent and ∼100–150 microns in its mediolateral extent. Yet, within this small, tightly packed space we found that biocytin-filled PBG cells exhibited a wide variety of morphologies. Since SC-PBG projections are organized in a topographic manner, the orientation of PBG dendrites is likely an important component of the construction of their receptive field properties. Therefore, using criteria similar to those used to categorize SC neurons (Gale and Murphy, 2014; Whyland et al., 2020), we divided the PBG neurons into four groups: stellate, asymmetrical, and narrow field (asymmetric vs. symmetric). In a previous study conducted in rats in which PBG neurons were also filled via biocytin, most filled neurons’ dendrites were oriented toward the lateral wall of the midbrain, with fewer cells having more of a dorsal-ventral orientation (“cylindrical shape”) (Goddard et al., 2007). Our biocytin-filled neurons showed a greater diversity of shapes and orientations, comparable to previous descriptions of rat PBG neurons based on Golgi staining techniques (Tokunaga and Otani, 1978). In this Golgi study, PBG neurons were also placed into four categories, which were similar to those we used: pyramidal (stellate), fusiform (stellate or narrow-field symmetrical), hemispheric (asymmetrical), and cylindrical (narrow-field, either type).

While we did not detect structure-function correlations in our study, the parameters that we measured were limited. PBG morphology may be related to projection target, a feature we did not investigate in the current study. The PBG projects to the ipsilateral pulvinar, and bilaterally to the dorsal lateral geniculate nucleus (dLGN), central amygdala, and SC (Diamond et al., 1992; Usunoff et al., 2007; Shang et al., 2015, 2018; Whyland et al., 2020; Sokhadze et al., 2022). Retrograde tracing studies have revealed that PBG neurons can branch to innervate several different contralateral targets, while ipsilateral projections arise from separate populations of PBG neurons (Sefton and Martin, 1984; Usunoff et al., 2006, 2007). Moreover, several studies have noted that PBG neurons that project to the contralateral SC are larger than those that project to the ipsilateral SC, and additionally that the PBG forms anterior/posterior subdivisions corresponding to these contralateral or ipsilateral projections, respectively (Watanabe and Kawana, 1979; Jen et al., 1984; Künzle and Schnyder, 1984; Baizer et al., 1991; Jiang et al., 1996; Deichler et al., 2020). Most PBG neurons express choline acetyl transferase (but not the vesicular acetylcholine transporter, VAChT; Sokhadze et al., 2022), and the type 2 vesicular glutamate transporter (vGlut2). However additional protein expression patterns may be limited to subsets of PBG neurons (Wang et al., 1988). Subsets of PBG neurons are also labeled in a variety of transgenic mouse lines including NTSR1-GN209-Cre and ChAT-Cre mice (Gale and Murphy, 2014; Sokhadze et al., 2022). Therefore, future studies may reveal that projection targets and/or protein expression patterns are related to features that we quantified in our characterization of PBG morphology.

### GABAergic projections to the PBG from non-tectal sources

Our electron microscopic analysis revealed that approximately 40% of the synaptic terminals in the PBG contain GABA, but that less than 10% of SC-PBG projections contain GABA. This discrepancy suggests that the PBG receives additional non-tectal sources of GABAergic input. In fact, we found that in GAD67-GFP mice, the PBG contains many GFP/vGAT-labeled puncta. Since we previously determined that SG-PBG neurons do not contain GFP in the GAD67-GFP line (Whyland et al., 2020), we conclude that these GFP-labeled puncta in the PBG do not originate from the SC.

The avian nucleus isthmi contains a subdivision of GABAergic neurons, the nucleus isthmi magnocellularis (Imc) which projects to the optic tectum as well as the cholinergic neurons of the nucleus isthmi parvocellularis (Ipc; Wang et al., 2004, 2006). The Imc has been proposed to provide global inhibition of the tectum and the Ipc as part of a proposed “stimulus selection network” that boosts the transmission of salient stimuli while suppressing non-relevant stimuli (Marín et al., 2007; Asadollahi et al., 2010; Asadollahi and Knudsen, 2016; Schryver and Mysore, 2019). Retrograde tracing studies in mammals have identified GABAergic neurons in the peri-parabigeminal area that project to the SC (Appell and Behan, 1990). If these GABAergic peri-parabigeminal neurons also project to the PBG, their function might be similar to that suggested for the avian Imc.

To test this possibility, we expressed channelrhodopsin in GABAergic neurons surrounding the PBG to determine whether their activation would inhibit PBG neurons. However, we found that projections from the tegmentum to the PBG were sparse and less than 10% of recorded PBG neurons responded to their activation. Thus, the PBG likely receives other non-tectal sources of inhibition. It is also possible that the PBG contains intrinsic GABAergic interneurons. In the GAD67-GFP line we identified a small number of GFP-labeled neurons within the PBG, and previous Golgi studies have suggested that PBG neurons might be divided into intrinsic neurons and projection neurons (Tokunaga and Otani, 1978). We did not identify any dendrodendritic connections in our electron microscopic images, and we did not identify any axon-like projections within the PBG originating from the neurons we filled with biocytin. However, thin axons are often difficult to fill with biocytin, axons projections may have exited our 300 μm thick slices, and if interneurons are sparse, they may not have been included in our sample. The GAD67-GFP, GAD2-cre and/or other mouse lines may be useful for future investigations of this previously unrecognized complexity of PBG circuits.

### Functional implications

As outlined in the Introduction, PBG neurons respond to looming and sweeping visual stimuli, exhibit direction-selective responses, and may further encode the saliency of signals to contribute to the initiation of appropriate behavioral responses (Sherk, 1978, 1979a; Ma et al., 2013; Shang et al., 2015, 2018; Zingg et al., 2017; Reinhard et al., 2019; Fernandes et al., 2021; Wang et al., 2022). In fact, optogenetic and chemogenetic manipulations of tectal inputs to the mouse PBG have been found to alter behavioral responses. Optogenetic activation of PBG inputs that originate from parvalbumin neurons initiate escape responses (Shang et al., 2015, 2018), while chemogenetic inactivation of NFV SC neurons that project to the PBG (in the Grp-KH288-cre line) impairs accurate orienting and pursuit in a prey capture paradigm which is dependent on binocular vision (Hoy et al., 2019; Berson, 2021; Johnson et al., 2021; Allen et al., 2022). Thus, there is substantial evidence that PBG circuits in the mouse play important roles in visually-guided behavior.

Our study revealed previously unappreciated complexities in the circuits of the PBG that likely impact the initiation and choice of behavioral responses. We found that most PBG neurons receive convergent excitatory and inhibitory inputs from the SC and that additional GABAergic inputs arise from non-tectal sources. Moreover, we found that the dendritic arbors of PBG vary widely in their orientations and extent. While it is still unclear how these circuit details impact PBG responses and subsequent animal behavior, they serve to direct future anatomical studies and may help to inform and interpret current and future functional studies.

## Data availability statement

The raw data supporting the conclusions of this article will be made available by the authors, without undue reservation.

## Ethics statement

This animal study was reviewed and approved by the University of Louisville Institutional Animal Care and Use Committee.

## Author contributions

KW: conceptualization, investigation, formal analysis, visualization, writing—original draft preparation, writing—review and editing, and funding acquisition. SM: investigation and formal analysis. AS: investigation, formal analysis, and visualization. MB: conceptualization, formal analysis, visualization, writing—review and editing, and funding acquisition. All authors contributed to the article and approved the submitted version.
